# HIV Dementia: A Bibliometric Analysis and Brief Review of the Top 100 Cited Articles

**DOI:** 10.7759/cureus.25148

**Published:** 2022-05-19

**Authors:** Taimoor Hussain, Andre Corraes, Khalida Walizada, Rajeswari Khan, Jafrikh Thamara Kunnath, Tuba Khan, Asjad Salman Zahid, Zahra Mushtaq, Mohit Bhagia, Vishnu R Bhure

**Affiliations:** 1 Neurology, Bolan Medical College, Quetta, PAK; 2 Department of Medicine and Surgery, Universidade Federal do Rio de Janeiro, Rio de Janeiro, BRA; 3 Department of Neurological Surgery, Hamad Medical Corporation, Doha, QAT; 4 Medicine and Surgery, Hospital College of Medicine and Sagore Dutta Hospital, Kolkata, IND; 5 Department of Internal Medicine, Government Medical College Kozhikode, Kozhikode, IND; 6 Medicine and Surgery, Ziauddin Medical College, Karachi, PAK; 7 Internal Medicine, Allama Iqbal Memorial Teaching Hospital, Lahore, PAK; 8 Internal Medicine, Sandeman Provincial Hospital, Quetta, PAK; 9 Neurology, Nanavati Max Super Speciality Hospital, Mumbai, IND; 10 Physical Therapy, Datta Meghe Institute of Medical Sciences, Wardha, IND

**Keywords:** review, hiv-associated dementia, hiv-associated neurocognitive disorders, bibliometric analysis, hand, had

## Abstract

Dementia is a syndrome of cognitive impairment that affects an individual’s ability to live independently. The number of people living with dementia worldwide in 2015 was estimated at 47.47 million. The American Academy of Neurology (AAN) criteria for human immunodeficiency virus (HIV)-associated dementia (HAD) require an acquired abnormality in at least two cognitive (non-motor) domains and either an abnormality in motor function or specified neuropsychiatric/psychosocial domains. HIV is the most common cause of dementia below 60 years of age.

Citation frequencies are commonly used to assess the scholarly impact of any scientific publication in bibliometric analyses. It helps depict areas of higher interest in terms of research frequency and trends of citations in the published literature and identify under-explored domains of any field, providing useful insight and guidance for future research avenues. We used the database “Web of Science” (WOS) to search for the top 100 cited articles on HIV-associated dementia. The keywords “HIV dementia” and “HIV-associated neurocognitive disorders” (HAND) were used. The list was generated by two authors after excluding articles not pertaining to HIV dementia. The articles were then assigned to authors to extract data to make tables and graphical representations. Finally, the manuscript was organized and written describing the findings of the bibliometric study.

These 100 most cited articles on HIV dementia were published between years 1986 and 2016. The highest number of the articles was from 1999 (n=9). The year 1993-2007 contributed consistently two publications to the list. The articles are from 42 journals, and among them, the Annals of Neurology (n=16) and the Journal of Neurology (n=15) published most of the articles. Justin C. McArthur with 25 publications contributed the highest number of papers to the list by any author. The USA collaborated in the highest number of publications (n=87). American institutes were leading the list with the most publications. The Johns Hopkins University collaborated on 37 papers. The most widely studied aspect of HIV dementia was pathogenesis. Incidence and prevalence, clinical features, and pre- and post-highly active antiretroviral therapy (HAART) era were also discussed in the articles.

Beyond America, the research should be expanded to low-income countries and those affected more by HIV. Therefore, other countries and their institutes should participate more in HIV-associated dementia research. Anticipating the rising resistance to existing antiretrovirals, we should develop new therapeutic options. There is room for research in many aspects of HIV dementia care.

## Introduction and background

Dementia is a syndrome of cognitive impairment or cognitive decline that affects an individual’s ability to live independently. Around 47 million had dementia in 2015, which could touch 135.46 million in 2050 [1]. With an annual 2.4 million deaths, dementia is the fifth leading cause of death worldwide [2]. According to the Rotterdam study, the main causes of dementia were Alzheimer’s disease (72%), followed by vascular dementia (16%), Parkinson’s disease dementia (6%), and other dementias (5%) [3]. Human immunodeficiency virus (HIV) dementia is one of the rare causes of dementia. However, HIV is the major cause of dementia below 60 years of age and the most common cause of dementia in young American adults [4,5].

There are a number of neurologic manifestations of HIV, which include but are not limited to encephalitis/meningitis by opportunistic infections, radiculopathies, myopathies, and neuropathies [6]. HAND is the acronym for HIV-associated neurocognitive disorders. According to the 1991 American Academy of Neurology (AAN) criteria, HIV-associated dementia (HAD) and minor cognitive motor disorder (MCMD) are two levels of neurologic manifestations of HIV. Briefly, the AAN criteria for HAD require an acquired abnormality in at least two cognitive (non-motor) domains and either an abnormality in motor function or specified neuropsychiatric/psychosocial domains. These abnormalities affect activities of daily living (ADLs). However, other differentials should be ruled out, and the patient’s mental state should permit assessment of these domains. Depending on which of the two from the second criteria were met in addition to the first criteria, the AAN further classified HAD into three subtypes: HAD with motor symptoms, HAD with behavioral or psychosocial symptoms, and HAD with both motor and behavioral/psychosocial symptoms [7].

Bibliometric analyses are commonly used to assess the scholarly impact of any scientific publication. Bibliometric analyses help depict areas of higher interest in terms of research, emerging trends, and collaboration patterns in the published literature and identify under-explored domains of any field, providing useful insight and guidance to funding authorities regarding the productive expenditure of resources. One of the approaches in the bibliometric analysis is citation frequency, which studies how frequently an article has been cited by investigators. Hence, the most cited article are considered academically significant. Articles with high citations can affect readership and consequently practice and future research. However, citation alone is not the sole comprehensive parameter for high-impact articles.

Materials and methods

The database “Web of Science” (WOS) was used to search for the top 100 cited articles on HIV-associated dementia. The keywords “HIV dementia” and “HIV-associated neurocognitive disorders” were used. All articles published up to September 2020 and earlier (since the indexing of WOS) were included. The list was generated from WOS by two authors after excluding articles not pertaining specifically to HIV dementia. Articles that discussed only HIV, other types of dementia, other types of central nervous system (CNS) infections, and other neurologic manifestations of HIV were also excluded. The articles were then assigned to authors to review, extract data, and record the data on an Excel datasheet. By generating and reviewing the 100 most cited articles, we compiled the list of top authors, institutions, years of publication, countries, types of study, and journals contributing significantly to HIV dementia research. The Excel data sheet was then used to make tables and graphical representations for visualization and comparison. Finally, the manuscript was organized and written describing the findings of the bibliometric study. An overview of the articles is also provided, highlighting some of the landmark findings of the top-cited articles.

Our research aims to explore which aspect of HIV dementia is the most studied and had a high impact based on the citation, ranging from clinical signs and pathogenesis to treatment. Such high-impact publications introduce potential areas of knowledge update for physicians and influence research areas for the future.

## Review

**Figure 1:**
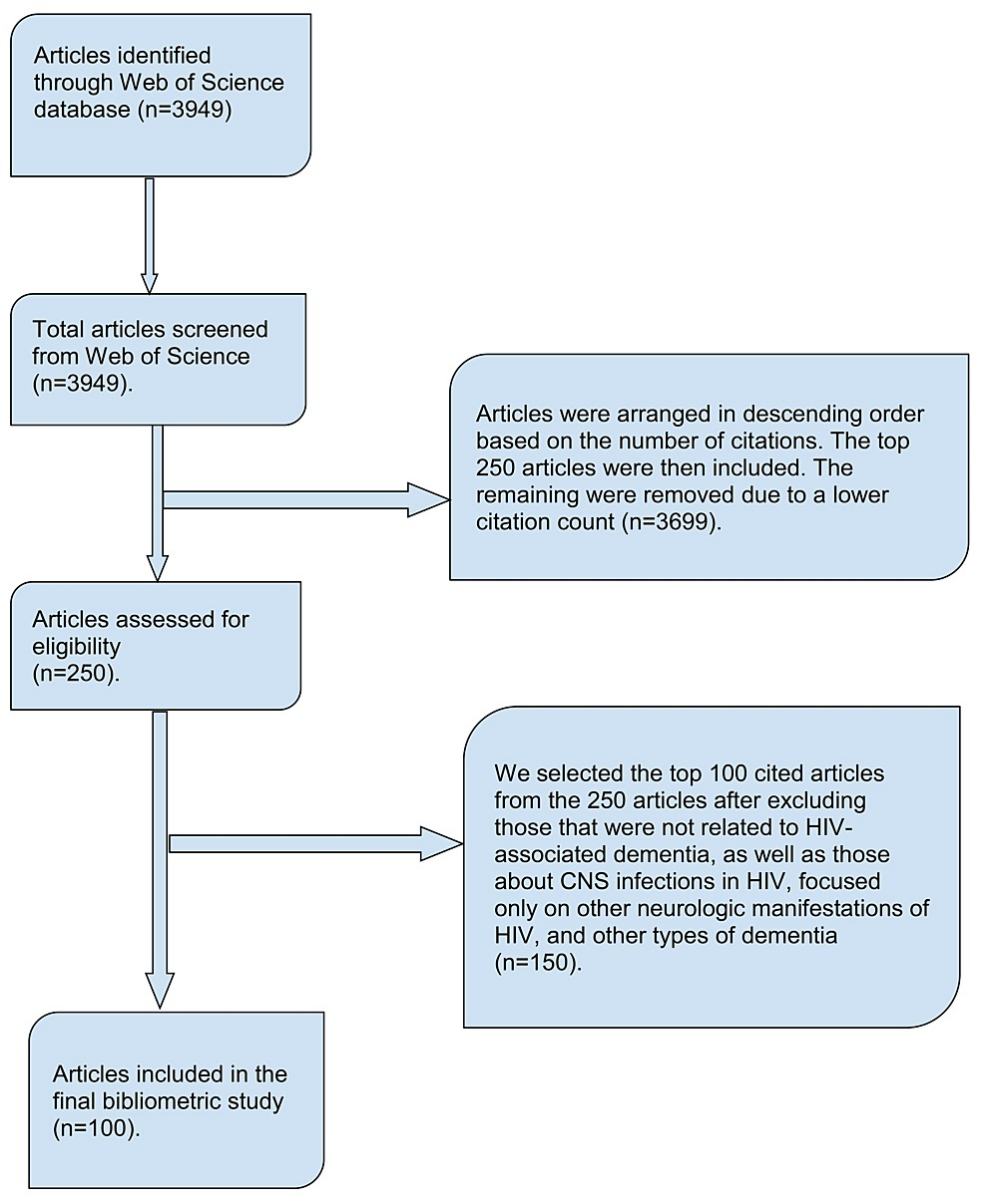
Search strategy and extraction of data from the Web of Science

Our search yielded 3949 publications from the beginning of WOS indexing up to September 2020. We arranged the articles based on citation count in descending order. After removing duplicates and articles not related to the topic as deemed by two independent reviewers, we selected the top 100 publications based on citation count for our analysis (Figure 1).

The articles were thoroughly studied and searched for data regarding citation count, year of publication, journals and their impact factor, authors and author pattern, institutions, and collaborating countries. The list of the top 100 articles based on citation count is shown in Table 1.

**Table 1:** Top 100 cited articles

No.	Citations	Research title
1	1425	Updated research nosology for HIV-associated neurocognitive disorders [7]
2	1257	HIV-associated neurocognitive disorders persist in the era of potent antiretroviral therapy [8]
3	1243	The brain in AIDS: central nervous system HIV-1 infection and AIDS dementia complex [9]
4	1220	The AIDS dementia complex: II. neuropathology [10]
5	914	Pathways to neuronal injury and apoptosis in HIV-associated dementia [11]
6	820	HIV-associated neurocognitive disorders before and during the era of combination antiretroviral therapy: differences in rates, nature, and predictors [12]
7	752	The neuropathogenesis of AIDs [13]
8	595	Nomenclature and research case definitions for neurologic manifestations of human immunodeficiency virus‐type 1 (HIV‐1) infection [14]
9	570	Central nervous system damage produced by expression of the HIV-1 coat protein GP120 in transgenic mice [15]
10	556	Immunocytochemical quantitation of human immunodeficiency virus in the brain: correlations with dementia [16]
11	545	Dementia in AIDS patients: incidence and risk factors [17]
12	456	Neuronal loss in the frontal cortex in HIV infection [18]
13	453	HIV-associated neurologic disease incidence changes: multicenter AIDS Cohort Study, 1990-1998 [19]
14	451	Induction of monocyte chemoattractant protein-1 in HIV-1 Tat-stimulated astrocytes and elevation in AIDS dementia [20]
15	445	Neocortical damage during HIV infection [21]
16	426	Dementia associated with the acquired immunodeficiency syndrome [22]
17	419	HIV-associated cognitive impairment before and after the advent of combination therapy [23]
18	409	Intracerebral cytokine messenger RNA expression in acquired immunodeficiency syndrome dementia [24]
19	402	The role of macrophage/microglia and astrocytes in the pathogenesis of three neurologic disorders: HIV-associated dementia, Alzheimer disease, and multiple sclerosis [25]
20	401	The prevalence and incidence of neurocognitive impairment in the HAART era [26]
21	397	Cognitive dysfunction in HIV patients despite long-standing suppression of viremia [27]
22	393	Chemokines and activated macrophages in HIV gp120-induced neuronal apoptosis [28]
23	376	Dendritic injury is a pathological substrate for human immunodeficiency virus-related cognitive disorders [29]
24	364	Immunologic NO synthase: elevation in severe AIDS dementia and induction by HIV-1 gp41 [30]
25	335	Spectrum of human immunodeficiency virus-associated neocortical damage [31]
26	321	Microbial translocation is associated with increased monocyte activation and dementia in AIDS patients [32]
27	309	Cerebral white matter changes in acquired immunodeficiency syndrome dementia: alterations of the blood-brain barrier [33]
28	308	Relationship between human immunodeficiency virus-associated dementia and viral load in cerebrospinal fluid and brain [34]
29	307	Changes to AIDS dementia complex in the era of highly active antiretroviral therapy [35]
30	299	HIV antigen in the brains of patients with the AIDS dementia complex [36]
31	298	Clinical-neuropathologic correlation in HIV-associated dementia [37]
32	294	Human immunodeficiency virus-associated dementia: an evolving disease [38]
33	291	In situ detection of polymerase chain reaction-amplified HIV-1 nucleic acids and tumor necrosis factor-alpha RNA in the central nervous system [39]
34	288	The epidemiology of human immunodeficiency virus-associated neurological disease in the era of highly active antiretroviral therapy [40]
35	285	Unique monocyte subset in patients with AIDS dementia [41]
36	284	Oxidative stress and the pathogenesis of neurodegenerative disorders [42]
37	280	Transient exposure to HIV-1 Tat protein results in cytokine production in macrophages and astrocytes. A hit and run phenomenon [43]
38	280	Human immunodeficiency virus-infected macrophages produce soluble factors that cause histological and neurochemical alterations in cultured human brains [44]
39	279	HIV dementia: an evolving disease [45]
40	272	Beta-chemokines MCP-1 and RANTES are selectively increased in cerebrospinal fluid of patients with human immunodeficiency virus-associated dementia [46]
41	271	Human immunodeficiency virus-associated neurocognitive disorders: mind the gap [47]
42	269	Higher frequency of dementia in older HIV-1 individuals: the Hawaii Aging with HIV-1 cohort [48]
43	269	Temporal trends in the incidence of HIV-1-related neurologic diseases: multicenter AIDS cohort study, 1985-1992 [49]
44	264	HIV infection and dementia [50]
45	264	HIV dementia scale: a rapid screening test [51]
46	258	CCL2/monocyte chemoattractant protein-1 mediates enhanced transmigration of human immunodeficiency virus (HIV)-infected leukocytes across the blood-brain barrier: a potential mechanism of HIV-CNS invasion and NeuroAIDS [52]
47	257	Human immunodeficiency virus type 1 infection alters chemokine beta peptide expression in human monocytes: implications for recruitment of leukocytes into brain and lymph nodes [53]
48	256	Cerebrospinal fluid human immunodeficiency virus type 1 RNA levels are elevated in neurocognitively impaired individuals with acquired immunodeficiency syndrome [54]
49	253	Identification and cloning of human astrocyte genes displaying elevated expression after infection with HIV-1 or exposure to HIV-1 envelope glycoprotein by rapid subtraction hybridization, RaSH [55]
50	251	CNS invasion by CD14+/CD16+ peripheral blood-derived monocytes in HIV dementia: perivascular accumulation and reservoir of HIV infection [56]
51	249	Reduced basal ganglia volume in HIV-1-associated dementia: results from quantitative neuroimaging [57]
52	248	Potential hazard of pharmacokinetic interactions between lopinavir-ritonavir protease inhibitors and irinotecan [58]
53	245	Abundant expression of HIV NEF and REV proteins in brain astrocytes in vivo is associated with dementia [59]
54	243	HIV-induced metalloproteinase processing of the chemokine stromal cell derived factor-1 causes neurodegeneration [60]
55	242	Human immunodeficiency virus (HIV) proteins in neuropathogenesis of HIV dementia [61]
56	242	Intracellular CXCR4 signaling, neuronal apoptosis and neuropathogenic mechanisms of HIV-1-associated dementia [62]
57	241	HIV-1 infection and AIDS: consequences for the central nervous system [63]
58	233	HIV-1 infection and AIDS dementia are influenced by a mutant MCP-1 allele linked to increased monocyte infiltration of tissues and MCP-1 levels [64]
59	224	CD4-independent, CCR5-dependent infection of brain capillary endothelial cells by a neurovirulent simian immunodeficiency virus strain [65]
60	216	Microglia express CCR5, CXCR4, and CCR3, but of these, CCR5 is the principal coreceptor for human immunodeficiency virus type 1 dementia isolates [66]
61	214	Extensive astrocyte infection is prominent in human immunodeficiency virus-associated dementia [67]
62	214	Plasma viral load and CD4 lymphocytes predict HIV-associated dementia and sensory neuropathy [68]
63	211	Human immunodeficiency virus encephalitis is the pathological correlate of dementia in acquired immunodeficiency syndrome [69]
64	209	AIDS dementia complex and HIV-1 brain infection: clinical-virological correlations [70]
65	206	Cell death in HIV dementia [71]
66	203	Elevated central nervous system prostaglandins in human immunodeficiency virus-associated dementia [72]
67	196	HIV-1 Tat through phosphorylation of NMDA receptors potentiates glutamate excitotoxicity [73]
68	196	Neurobiological aspects of human immunodeficiency virus infection: neurotoxic mechanisms [74]
69	189	HIV-infected subjects with the E4 allele for APOE have excess dementia and peripheral neuropathy [75]
70	188	Persistence of HIV-associated cognitive impairment, inflammation, and neuronal injury in era of highly active antiretroviral treatment [76]
71	188	Neurological complications of HIV infection [77]
72	187	Perturbation of sphingolipid metabolism and ceramide production in HIV-dementia [78]
73	185	Distribution of brain HIV load in AIDS [79]
74	184	Dementia and neurocognitive disorders due to HIV-1 infection [80]
75	182	Incidence and prevalence of neurological disorders associated with HIV since the introduction of highly active antiretroviral therapy (HAART) [81]
76	180	The Tat protein of HIV-1 induces tumor necrosis factor-alpha production. Implications for HIV-1-associated neurological diseases [82]
77	180	Levels of human immunodeficiency virus type 1 RNA in cerebrospinal fluid correlate with AIDS dementia stage [83]
78	173	Cerebral metabolite abnormalities correlate with clinical severity of HIV-1 cognitive motor complex [84]
79	171	Neuronal apoptosis does not correlate with dementia in HIV infection but is related to microglial activation and axonal damage [85]
80	165	Role of the pro-inflammatory cytokines TNF-alpha and IL-1beta in HIV-associated dementia [86]
81	163	Evidence for a change in AIDS dementia complex in the era of highly active antiretroviral therapy and the possibility of new forms of AIDS dementia complex [87]
82	163	Highly active antiretroviral therapy reverses brain metabolite abnormalities in mild HIV dementia [88]
83	162	Human immunodeficiency virus type 1 Vpr induces apoptosis in human neuronal cells [89]
84	160	Mechanisms of the blood-brain barrier disruption in HIV-1 infection [90]
85	160	Neurocognitive impairment is an independent risk factor for death in HIV infection [91]
86	158	Cerebrospinal fluid levels of MMP-2, 7, and 9 are elevated in association with human immunodeficiency virus dementia [92]
87	156	Oxidative stress in HIV demented patients and protection ex vivo with novel antioxidants [93]
88	155	Evaluation of HIV RNA and markers of immune activation as predictors of HIV-associated dementia [94]
89	155	Marked improvement in survival following AIDS dementia complex in the era of highly active antiretroviral therapy [95]
90	155	Cerebrospinal fluid HIV RNA originates from both local CNS and systemic sources [96]
91	154	Neuronal apoptosis induced by HIV-1 Tat protein and TNF-alpha: potentiation of neurotoxicity mediated by oxidative stress and implications for HIV-1 dementia [97]
92	154	Neurologic manifestations of HIV infection [6]
93	154	A review of neuronal damage in human immunodeficiency virus infection: its assessment, possible mechanism and relationship to dementia [98]
94	153	Neurotoxicity and dysfunction of dopaminergic systems associated with AIDS dementia [99]
95	152	HIV-associated neurocognitive disorder: pathogenesis and therapeutic opportunities [100]
96	152	Decreased brain dopaminergic transporters in HIV-associated dementia patients [101]
97	151	HIV dementia: the role of the basal ganglia and dopaminergic systems [102]
98	148	Plasma concentration of the neurofilament light protein (NFL) is a biomarker of CNS injury in HIV infection: a cross-sectional study [103]
99	148	Oxidative stress and neuroAIDS: triggers, modulators and novel antioxidants [104]
100	145	Neurotoxicity of HIV-1 proteins gp120 and Tat in the rat striatum [105]

Year of publication

**Figure 2:**
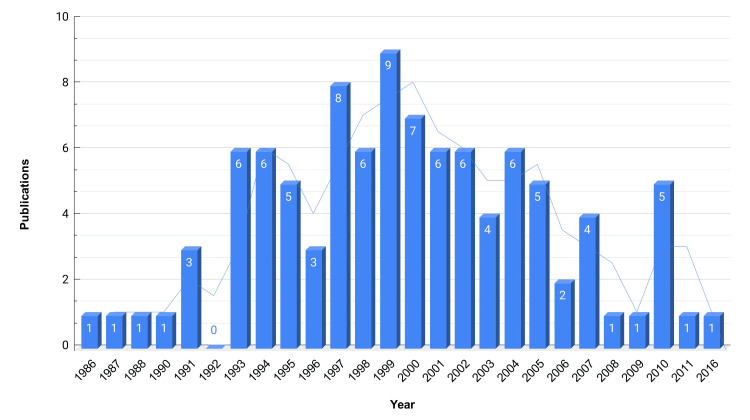
Number of publications versus years with trend line

Articles making it to the list of top-cited articles on HIV dementia were published from the year 1986 to 2016. More than two-thirds of the articles were published in the first half of the 31 years. Further details are presented in Figure 2.

Journals, number of publications, and impact factor

Three journals (Annals of Neurology (n=18), Neurology (n=15), and AIDS (n=8)) were responsible for more than one-third of all publications. The list also includes many high impact factor journals. At least one paper each from 42 different journals made it to the list of top 100 cited articles. Further details are shown in Table 2.

**Table 2:** Top journals list

No.	Journals	Number of publication	Impact factor of journals (JCR 2020)
1	Annals of Neurology	18	9.037
2	Neurology	15	8.770
3	AIDS	8	4.511
4	Journal of Neurovirology	6	2.354
5	Proceedings of the National Academy of Sciences of the United States of America	5	9.412
6	Science	3	41.845
7	The Lancet	3	60.392
8	Journal of Biological Chemistry	2	4.238
9	Journal of Neuroimmunology	2	3.033
10	The Journal of Infectious Diseases	2	5.022
11	Journal of Virology	2	4.501
12	Cell Death and Differentiation (Nature)	2	10.717
13	Nature	2	42.778
14	Journal of Psychopharmacology	2	3.121
15	Journal of Neurochemistry	1	4.066
16	Progress in Neurobiology	1	9.371
17	Nature Medicine	1	36.130
18	Brain Pathology	1	5.568
19	Seminars in Neurology	1	2.034
20	Journal of Neurology, Neurosurgery and Psychiatry	1	8.234
21	Neuropathology and Applied Neurobiology	1	7.500
22	European Journal of Clinical Investigation	1	3.481
23	Cellular and Molecular Neurobiology	1	3.606
24	JAMA Neurology	1	13.608
25	Journal of Neurovirology	1	2.354
26	Annals of Internal Medicine	1	21.317
27	Journal of Neuropathology & Experimental Neurology	1	2.923
28	Journal of Neurochemistry	1	4.066
29	Journal of Neuroimmune Pharmacology	1	4.113
30	Brain Research	1	2.733
31	Brain	1	11.337
32	eBioMedicine	1	5.736
33	Trends in Neurosciences	1	12.891
34	Nature Reviews Immunology	1	40.358
35	The New England Journal of Medicine	1	74.699
36	Journal of the Neurological Sciences	1	3.115
37	The American Journal of Pathology	1	3.491
38	PLOS One	1	2.740
39	International Review of Neurobiology	1	2.627
40	Journal of Acquired Immune Deficiency Syndrome and Human Retrovirology	1	3.863
41	The Journal of Neuroscience	1	5.673
42	Journal of Clinical Investigation	1	11.864

Institutions and their number of records

The Johns Hopkins University was the single institution with the highest number of publications (n=37), publishing twice as much as the institute with the second-highest number of papers (University of California San Diego, n=14). We limited our list to institutes that contributed more than three publications to the list. American institutes lead the list. The list includes other prestigious institutions such as the University of California (San Diego, Los Angeles, and San Francisco), Columbia, and Harvard University. Further details are presented in Table 3.

**Table 3:** Institutions and their number of publications

Institutions	Number of publications
Johns Hopkins University (USA)	37
University of California San Diego (USA)	14
University of Kentucky (USA)	8
St. Vincent’s Hospital (Australia)	7
University of California Los Angeles (USA)	7
University of Manitoba (Canada)	7
Harvard University (USA)	6
Mount Sinai New York (Icahn School of Medicine/Mount Sinai Medical Center)	6
North Western University (Chicago, USA)	6
University of Pittsburgh (USA)	6
University of New South Wales (Australia)	6
National Institute of Health (USA)	5
University of California San Francisco (USA)	5
University of Nebraska (USA)	5
University of North Carolina (USA)	4
University of Pennsylvania (USA)	4
University of Washington (USA)	4

Authors and their number of publications

We limited our list to those authors who contributed five or more publications. Justin C. McArthur leads the list, and 28 of his publications made it to the list. The other highly published authors and their h-index are presented in Table 4.

**Table 4:** Authors and their number of publications

Author name	Number of publications	h-index of authors (Scopus)
Justin C. McArthur	28	106
Avindra Nath	12	91
Ned Charlton Sacktor	10	56
Ola A. Selnes	10	71
Bruce James Brew	8	75
Ronald Joseph Ellis	8	75
Igor Grant	7	97
Richard W. Price	7	74
Bruce Arnold Cohen	6	58
Christopher Power	6	61
J.A. McCutchan	6	72
Robert K. Heaton	6	100
Steven L. Wesselingh	6	50
C.A. Willey	5	59
Eric N. Miller	5	50
Howard E. Gendelman	5	92
Ian Abramson	5	44
J. Hampton Atkinson	5	58
James Thompson Becker	5	95
Jonathan D. Glass	5	77

Countries and their number of publications

America with 87 publications leads the list. Other collaborating countries are presented in Table 5.

**Table 5:** Collaborating countries and number of publications

Country	Number of publications
USA	87
Australia	8
Canada	8
UK	4
Germany	3
Sweden	3
Netherlands/France/Italy	2

Author pattern

**Figure 3:**
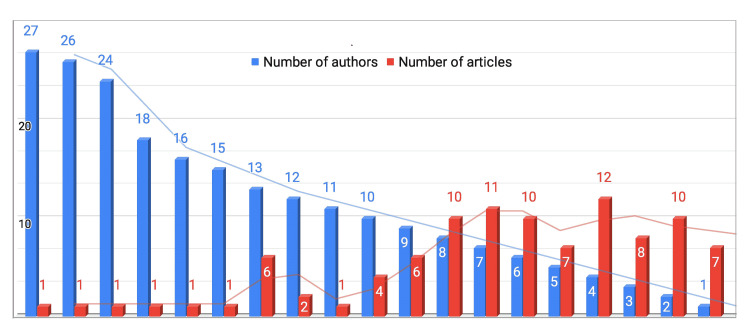
Author pattern in the articles

With 27 authors and two groups, the article “HIV-associated neurocognitive disorders before and during the era of combination antiretroviral therapy: differences in rates, nature, and predictors” had the highest number of authors in any publication. There were seven papers written by a single author. The trend lines for the number of authors and articles display an inverse relation to each other as shown in Figure 3. We did not count the collaborating author groups in making this list, as the groups alone were comprised of many authors, with some of the authors mentioned in both the groups and the main author list of the article. The groups include the “CHARTER Group” and “HIV Neurobehavioral Research Center (HNRC) Group.”

HIV dementia main topics and review

The papers were classified by counting the frequency of their keywords, reading their full content, and defining their themes. Articles were primarily classified into experimental studies, observational studies or reviews, and one report. The pathogenesis of HIV-associated neurocognitive impairment (NCI) was the most discussed aspect in experimental studies, observational studies, and reviews. However, other aspects such as treatment, diagnosis, risk factors, incidence, prevalence, and clinical aspects were also explored. Most of the reviews discussed more than one aspect of HIV dementia, and all were counted in making the list. Further details of the classification of articles are represented in Table 6 and Table 7.

**Table 6:** Topics covered by review articles The sum of the percentages is more than 100% because some articles reviewed more than one aspect of HIV dementia, and there was considerable overlap.

Review articles	Number of articles with percentage
Review of diagnosis	4 (14%)
Review of epidemiology	6 (21%)
Review of pathogenesis	24 (85%)
Review of treatment	10 (35%)
Review of clinical aspects	6 (21%)

**Table 7:** Topics of observational studies The sum of the percentages is more than 100% because some articles discussed more than one aspect of HIV dementia, and there was considerable overlap.

Observational studies	Number of articles and percentage
Pathogenesis	39 (72%)
Incidence/prevalence	8 (14%)
Treatment	6 (11%)
Diagnosis	3 (5%)
Clinical aspects	2 (3%)
Risk factors	2 (3%)

We reviewed the top 100 cited articles that were published between 1986 and 2020. The first paper (published in 1986) discussed the pathological findings in subcortical white matter in the autopsy of 70 adult AIDS patients. The article concluded that “abnormalities were found predominantly in the white matter and in subcortical structures, with relative sparing of the cortex. Their frequency and severity generally correlated well with the degree and duration of clinical dementia” [10]. The last article (published in 2016) explored the plasma versus CSF concentration of neurofilament light protein (NFL) as a biomarker of CNS injury in HIV infection. “The pattern of NFL changes were almost identical in plasma and CSF, both exhibiting similar age-related increases in concentrations along with highest values in HAD” [103]. No articles from 2017 to 2020 made it to the list, probably due to the time required for particles to accumulate citations. The years 1999 (nine publications), 1997 (eight publications), and 2000 (seven publications) had the first, second, and third highest number of publications as per our list. The year 1993-2007 can be considered as the most productive and contributed a minimum of two publications to the list of the top 100 cited articles. Pathogenesis was consistently one of the most discussed aspects of HIV dementia.

The articles were published in 42 different journals. The top three journals that contributed more than one-third of the articles are “Annals of Neurology” with an impact factor (IF) of 9.037 (n=16), “Journal of Neurology” with an impact factor of 8.77 (n=15), and “AIDS” with an impact factor of 4.51 (n=8). Thus, authors tend to publish in journals dedicated to the specialty. All top six journals were dedicated to neurology, brain, or AIDS. The journals with the highest impact factors in the list are “The New England Journal of Medicine” (IF=74.69), followed by “The Lancet” (IF=60.392), “Nature” (IF=42.778), and “Science” (IF=41.845).

The 100 most cited articles were published by 42 different institutes. The Johns Hopkins University collaborated on 37 of the most cited article list. The Johns Hopkins University is consistently ranked number one in neurology globally [106]. The top six institutions are from the USA.

The 100 most cited articles were collaborated by 400+ different authors. The top three authors are Justin C. McArthur (n=28), Avindra Nath (n=12), and Ned Charlton Sacktor and Ola A. Selnes (n=10). According to the Johns Hopkins website, “Dr. Justin C. McArthur is well known for his research in HIV infection, multiple sclerosis, and other immune-mediated neurologic disorders. Dr. McArthur is the director of the Johns Hopkins/National Institute of Mental Health Research Center for Novel Therapeutics of HAND which aims to utilize our understanding of the disease pathophysiology for the same purpose” [107].

Among the collaborating countries, the USA has the highest number of contributions (n=87). According to the UNESCO science report, “US is the world leader in medical research, with 51% of federal research dedicated to life sciences and 46% to research worldwide [108]. In 2013, US pharmaceutical companies spent 40 billion dollars on R&D in America [108]. The National Institute of Health budget was around $30 billion in 2015” [108]. This explains why America is leading the list in our study as well.

The most widely studied aspect of HIV dementia was pathogenesis. Most of the articles about pathogenesis were observational case-control, cross-sectional, comparative, retrospective, or prospective cohort studies. A fair number of papers were reviews or experimental studies about pathogenesis and other aspects.

An excellent most recent review of pathogenesis from our list of articles is given by Lindl et al., which is summarized as follows: “HIV mainly infects macrophage lineage through a ‘Trojan Horse’ method. It crosses the blood-brain barrier (BBB) through infected monocytes that later differentiate into macrophages. As HIV cannot infect neurons directly, the direct and indirect model explains the pathogenesis in HAND. The direct model suggests that infected monocyte-derived cells release viral protein which interacts with neurons and leads to neuronal damage and death of neurons. The indirect or ‘bystander’ model suggests that neuronal death is initiated by the inflammatory response of the infected and uninfected non-neuronal cells against HIV infection and against HIV proteins released by directly infected cells. These two models together explain the pathogenesis. The indirect model is centered upon soluble factors including the viral proteins, glycoprotein 120, arachidonic acids, matrix metalloproteases, chemokines, growth factors, and pro-inflammatory cytokines, including tumor necrosis factor (TNF) to name a few. Astrocytes have neuroprotective functions which are disrupted by TNF and NO. TNF and NO also interfere with glutamate reuptake and BBB, while also resulting in more apoptosis in astrocytes. Diminished reuptake of glutamate and production of more stimulatory amino acids results in higher activation of NMDAR, raising the calcium level inside neurons to toxic levels, resulting in more free radicals and consequent death of neurons. Synaptic disruption and impairment of neurogenesis are some of the other observed neuropathologies.” [100]. The findings of some of the other highly cited articles are briefly discussed as follows. One study concluded that “blood-brain barrier (BBB) disruption leads to HIV dementia rather than demyelination, evidence suggesting the role of MMP (2, 7, and 9) targeting laminin, entactin, and collagen type IV. These are blood-brain barrier proteins, and they are also seen in higher quantities in the cerebrospinal fluid (CSF) of HIV dementia patients” [33,92]. HIV transactivator of transcription (Tat protein), viral protein R (Vpr protein), HIV Nef and Rev protein, and astrocytes’ role in pathogenesis are also well researched [43,55,59,73]. Articles exploring HIV Tat proteins, such as neuronal cytokine production (including TNF) and apoptosis induced by HIV tat proteins [43], and NMDA phosphorylation by HIV tat protein potentiating glutamate excitotoxicity are also among the highly cited articles [73]. Astrocytes are involved in maintaining the microenvironment around synapses and in transmitting signals across neurons. Therefore, a study exploring human astrocyte genes that were increasingly expressed in HIV infection received a high number of citations as well [55]. Genetic studies also made it to the list [64,75]. According to one study, the “MCP-1 -2578G allele reduced the risk of acquiring HIV by 50%, but during infection, it was associated with increased disease progression and risk of HAD” [64]. On the other hand, the E4 allele for APOE increased the risk of dementia and peripheral neuropathy [75].

The availability of dopamine transporters (DATs), CSF studies, biomarkers of CNS injury, and basal ganglia’s involvement in HIV dementia also made it to our list. Here, we discuss them briefly. In one study, it was suggested that “HIV dementia is subcortical as there is a selective atrophy of basal ganglia which explains the clinical spectrum” [57]. Unlike dopamine receptors, DAT is significantly deceased in HIV dementia patients as compared to seronegative controls, so HIV viral burden and DAT are inversely related [101]. Cerebrospinal fluid (CSF) neurofilament light chain (NFL) level is a biomarker of CNS injury in HIV dementia. However, in patients unwilling to undergo lumbar puncture, plasma (NFL) is just as reliable [103]. CSF studies also reported an increase in HIV RNA levels in AIDS patients [54]. Other CSF studies reported the correlation of viral load in CSF with HIV dementia and selective increase of chemokines such as beta-chemokine MCP-1 and RANTES in CSF of HIV dementia patients [46].

As per our list of the top 100 cited articles based on citation, the first article on antiretroviral therapy is published in 1999. Few other articles discussing antiretroviral therapy, pre- and post-highly active antiretroviral therapy (HAART) era, made it to the list. While some asserted the benefits of HAART, others discussed the disadvantages, differences, and new challenges of the post-HAART era. A brief summary of the findings of the selected articles is as follows. In their study, Heaton et al. estimated that the “prevalence of specific HAND diagnosis was 33% for asymptomatic NCI, 12% for mild NCI, and only 2% for HAD” [8]. An Australian study of the efficacy of HAART against AIDS dementia complex (ADC) versus other AIDS-defining illnesses (ADI) from 1992 to 1997 suggested that “HAART has more impact on ADIs as compared to ADC, as evidenced by the higher frequency of ADC relative to other ADIs” [35]. In their study of HAND pre-combination antiretroviral therapy (CART) and CART era, Heaton et al. concluded that “neurocognitive impairment (NCI) was associated with low nadir CD4 in both eras.” Therefore, early initiation of treatment may be beneficial in terms of preventing HAND due to sustained immunity. On the other hand, the “degree of current immunosuppression, estimated duration of infection, and viral suppression in CSF (on treatment) were related to deficits only pre-CART era. Disruptions in cognitive speed, motor skills, and verbal fluency were more common in the pre-CART era, while memory and executive dysfunctions were observed more in the CART era” [12]. One of the studies suggested that “as evident by impairments in verbal/visual memory and psychomotor/motor speed, HIV-associated NCI is still common in HIV patients with advanced stage, even after 1996 when HAART was introduced” [23], perhaps because “HIV-associated NCI and brain injury continue in the setting of chronic and stable disease’’ [76,8]. “No correlation was found between virological/immunological indicators and NCI. Only previous advanced immunosuppression and NCI were found to be correlated according to a study by Robertson et al.” [8]. However, Chang et al. reported the benefits of HAART “in terms of improvement in HIV cognitive motor complex (HIV-CMC), in terms of increased CD4 count, improvement in HIV dementia scale, and decrease in HI-CMC stage, In addition to improvement in systemic measures of HIV infection and improvement of brain injury measured by cerebral metabolites (myoinositol/creatine and choline/creatine), particularly the glial marker” [88]. Similarly, the advantages of HAART were supported in the study of the period 1995-1998 by Maschke et al. “in terms of lower incidence and prevalence of HIV dementia, polyneuropathy, and encephalitis, probably due to improved immunity or their direct CNS effects” [81]. Another study reported considerable improvement in survival after ADC in the era of HAART, despite the proportional increase in ADC at AIDS diagnosis [95]. One article suggested that patients live longer due to HAART with inactive and chronic forms of ADC. It is now weakly related to the CD4 cell count. Patients are prone to the possible cognitive effects of unappreciated abnormalities such as increased triglycerides and cholesterol levels, testosterone deficiency, and mitochondrial toxicity. There is considerable evidence suggesting an increased risk of Alzheimer’s disease, which will take time to verify [87]. The introduction of HAART in 1996 has considerably decreased the incidence of opportunistic infections and HIV dementia in developed countries. On the contrary, the incidence of toxic neuropathy has increased, and HIV dementia is emerging as a major cause of dementia in developing countries due to inadequate access to antiretroviral therapies [40].

The authors also expressed their concerns that HAND may increase due to resistance mutations to antiretroviral drugs. New treatments such as fusion inhibitors or immunomodulatory therapies may work as a short-term solution, as resistance will also develop against them with the passage of time [40]. Present antiretrovirals are not so effective in crossing the BBB. Therefore, “future studies should investigate chemokines and cytokine receptor antagonists, NMDA receptor blockers, voltage-activated calcium channel antagonists or non-NMDA glutamate antagonists, caspase inhibitors, and antioxidants as other therapies that may be useful” [11]. Drugs targeting HIV viral proteins such as “Tat” and “Vpr” are potential candidates as they have a role in the pathogenesis of HIV dementia.

Certainly, there always remains the need for further research. The leader in HIV dementia research, Justin C. McArthur, suggested that “in future research, clinical trials should explore the impact of the new antiretrovirals on NCI, including in backward regions with high HIV burden. The role of genetic polymorphisms, new biomarkers for prediction or identification of different types of HIV-D, in treatment responses and as risk factors, also needs further investigation” [45].

Limitation

Bibliometric analysis has its own limitations. Self-citation, relying on a single database, and missing older articles not indexed in the database can create bias in the bibliometric analysis. In addition, some of the recent publications will obviously take time to accumulate citations despite being high impact; thus, they might not yet have made it to the list.

## Conclusions

America and its institutions made major contributions to HIV dementia research. Pathogenesis was the most studied aspect. The most cited articles were published in high impact factor journals dedicated to neurology specialty. Incidence and prevalence, clinical features, the main pathways taking part in the pathogenesis of HIV of dementia, difference, and the benefits of the pre- and post-HAART era were explored. New and novel therapeutic options should be explored as resistance develops to existing antiretrovirals. Much of the research is confined to developed countries and conducted by America. It is felt that other countries, especially the low-income countries and those affected more by HIV, should encourage research culture to fill the void of regional demographic, clinicopathologic, and epidemiologic data. Regional research data can highlight local variations and perhaps modify all medical interventions tailored to specific regions.

## References

[1] Alzheimer’s Disease International (2013). Policy brief for G8 heads of government: the global impact of dementia 2013-2050. https://www.alzint.org/u/2020/08/GlobalImpactDementia2013.pdf.

[2] GBD 2016 Dementia Collaborators (2019). Global, regional, and national burden of Alzheimer's disease and other dementias, 1990-2016: a systematic analysis for the Global Burden of Disease Study 2016. Lancet Neurol.

[3] Ott A, Breteler MM, van Harskamp F, Claus JJ, van der Cammen TJ, Grobbee DE, Hofman A (1995). Prevalence of Alzheimer's disease and vascular dementia: association with education. The Rotterdam study. BMJ.

[4] Janssen RS (1992). Epidemiology of human immunodeficiency virus infection and the neurologic complications of the infection. Semin Neurol.

[5] McArthur JC, Sacktor N, Selnes O (1999). Human immunodeficiency virus-associated dementia. Semin Neurol.

[6] Simpson DM, Tagliati M (1994). Neurologic manifestations of HIV infection. Ann Intern Med.

[7] Antinori A, Arendt G, Becker JT (2007). Updated research nosology for HIV-associated neurocognitive disorders. Neurology.

[8] Heaton RK, Clifford DB, Franklin DR Jr (2010). HIV-associated neurocognitive disorders persist in the era of potent antiretroviral therapy: CHARTER study. Neurology.

[9] Price RW, Brew B, Sidtis J, Rosenblum M, Scheck AC, Cleary P (1988). The brain in AIDS: central nervous system HIV-1 infection and AIDS dementia complex. Science.

[10] Navia BA, Cho ES, Petito CK, Price RW (1986). The AIDS dementia complex: II. Neuropathology. Ann Neurol.

[11] Kaul M, Garden GA, Lipton SA (2001). Pathways to neuronal injury and apoptosis in HIV-associated dementia. Nature.

[12] Heaton RK, Franklin DR, Ellis RJ (2011). HIV-associated neurocognitive disorders before and during the era of combination antiretroviral therapy: differences in rates, nature, and predictors. J Neurovirol.

[13] González-Scarano F, Martín-García J (2005). The neuropathogenesis of AIDS. Nat Rev Immunol.

[14] (1991). Nomenclature and research case definitions for neurologic manifestations of human immunodeficiency virus‐type 1 (HIV‐1) infection. Report of a Working Group of the American Academy of Neurology AIDS Task Force. Neurology.

[15] Toggas SM, Masliah E, Rockenstein EM, Rall GF, Abraham CR, Mucke L (1994). Central nervous system damage produced by expression of the HIV-1 coat protein gp120 in transgenic mice. Nature.

[16] Glass JD, Fedor H, Wesselingh SL, McArthur JC (1995). Immunocytochemical quantitation of human immunodeficiency virus in the brain: correlations with dementia. Ann Neurol.

[17] McArthur JC, Hoover DR, Bacellar H (1993). Dementia in AIDS patients: incidence and risk factors. Neurology.

[18] Everall IP, Luthert PJ, Lantos PL (1991). Neuronal loss in the frontal cortex in HIV infection. Lancet.

[19] Sacktor N, Lyles RH, Skolasky R (2001). HIV-associated neurologic disease incidence changes: multicenter AIDS Cohort Study, 1990-1998. Neurology.

[20] Conant K, Garzino-Demo A, Nath A (1998). Induction of monocyte chemoattractant protein-1 in HIV-1 Tat-stimulated astrocytes and elevation in AIDS dementia. Proc Natl Acad Sci U S A.

[21] Wiley CA, Masliah E, Morey M (1991). Neocortical damage during HIV infection. Ann Neurol.

[22] Lipton SA, Gendelman HE (1995). Seminars in medicine of the Beth Israel Hospital, Boston. Dementia associated with the acquired immunodeficiency syndrome. N Engl J Med.

[23] Sacktor N, McDermott MP, Marder K (2002). HIV-associated cognitive impairment before and after the advent of combination therapy. J Neurovirol.

[24] Wesselingh SL, Power C, Glass JD (1993). Intracerebral cytokine messenger RNA expression in acquired immunodeficiency syndrome dementia. Ann Neurol.

[25] Minagar A, Shapshak P, Fujimura R, Ownby R, Heyes M, Eisdorfer C (2002). The role of macrophage/microglia and astrocytes in the pathogenesis of three neurologic disorders: HIV-associated dementia, Alzheimer disease, and multiple sclerosis. J Neurol Sci.

[26] Robertson KR, Smurzynski M, Parsons TD (2007). The prevalence and incidence of neurocognitive impairment in the HAART era. AIDS.

[27] Simioni S, Cavassini M, Annoni JM (2010). Cognitive dysfunction in HIV patients despite long-standing suppression of viremia. AIDS.

[28] Kaul M, Lipton SA (1999). Chemokines and activated macrophages in HIV gp120-induced neuronal apoptosis. Proc Natl Acad Sci U S A.

[29] Masliah E, Heaton RK, Marcotte TD (1997). Dendritic injury is a pathological substrate for human immunodeficiency virus-related cognitive disorders. HNRC Group. The HIV Neurobehavioral Research Center. Ann Neurol.

[30] Adamson DC, Wildemann B, Sasaki M (1996). Immunologic NO synthase: elevation in severe AIDS dementia and induction by HIV-1 gp41. Science.

[31] Masliah E, Achim CL, Ge N, DeTeresa R, Terry RD, Wiley CA (1992). Spectrum of human immunodeficiency virus-associated neocortical damage. Ann Neurol.

[32] Ancuta P, Kamat A, Kunstman KJ (2008). Microbial translocation is associated with increased monocyte activation and dementia in AIDS patients. PLoS One.

[33] Power C, Kong PA, Crawford TO, Wesselingh S, Glass JD, McArthur JC, Trapp BD (1993). Cerebral white matter changes in acquired immunodeficiency syndrome dementia: alterations of the blood-brain barrier. Ann Neurol.

[34] McArthur JC, McClernon DR, Cronin MF, Nance-Sproson TE, Saah AJ, St Clair M, Lanier ER (1997). Relationship between human immunodeficiency virus-associated dementia and viral load in cerebrospinal fluid and brain. Ann Neurol.

[35] Dore GJ, Correll PK, Li Y, Kaldor JM, Cooper DA, Brew BJ (1999). Changes to AIDS dementia complex in the era of highly active antiretroviral therapy. AIDS.

[36] Pumarola-Sune T, Navia BA, Cordon-Cardo C, Cho ES, Price RW (1987). HIV antigen in the brains of patients with the AIDS dementia complex. Ann Neurol.

[37] Glass JD, Wesselingh SL, Selnes OA, McArthur JC (1993). Clinical-neuropathologic correlation in HIV-associated dementia. Neurology.

[38] McArthur JC, Haughey N, Gartner S, Conant K, Pardo C, Nath A, Sacktor N (2003). Human immunodeficiency virus-associated dementia: an evolving disease. J Neurovirol.

[39] Nuovo GJ, Gallery F, MacConnell P, Braun A (1994). In situ detection of polymerase chain reaction-amplified HIV-1 nucleic acids and tumor necrosis factor-alpha RNA in the central nervous system. Am J Pathol.

[40] Sacktor N (2002). The epidemiology of human immunodeficiency virus-associated neurological disease in the era of highly active antiretroviral therapy. J Neurovirol.

[41] Pulliam L, Gascon R, Stubblebine M, McGuire D, McGrath MS (1997). Unique monocyte subset in patients with AIDS dementia. Lancet.

[42] Reynolds A, Laurie C, Mosley RL, Gendelman HE (2007). Oxidative stress and the pathogenesis of neurodegenerative disorders. Int Rev Neurobiol.

[43] Nath A, Conant K, Chen P, Scott C, Major EO (1999). Transient exposure to HIV-1 Tat protein results in cytokine production in macrophages and astrocytes. A hit and run phenomenon. J Biol Chem.

[44] Pulliam L, Herndier BG, Tang NM, McGrath MS (1991). Human immunodeficiency virus-infected macrophages produce soluble factors that cause histological and neurochemical alterations in cultured human brains. J Clin Invest.

[45] McArthur JC (2004). HIV dementia: an evolving disease. J Neuroimmunol.

[46] Kelder W, McArthur JC, Nance-Sproson T, McClernon D, Griffin DE (1998). Beta-chemokines MCP-1 and RANTES are selectively increased in cerebrospinal fluid of patients with human immunodeficiency virus-associated dementia. Ann Neurol.

[47] McArthur JC, Steiner J, Sacktor N, Nath A (2010). Human immunodeficiency virus-associated neurocognitive disorders: mind the gap. Ann Neurol.

[48] Valcour V, Shikuma C, Shiramizu B (2004). Higher frequency of dementia in older HIV-1 individuals: the Hawaii Aging with HIV-1 cohort. Neurology.

[49] Bacellar H, Muñoz A, Miller EN (1994). Temporal trends in the incidence of HIV-1-related neurologic diseases: multicenter AIDS cohort study, 1985-1992. Neurology.

[50] Gartner S (2000). HIV infection and dementia. Science.

[51] Power C, Selnes OA, Grim JA, McArthur JC (1995). HIV dementia scale: a rapid screening test. J Acquir Immune Defic Syndr Hum Retrovirol.

[52] Eugenin EA, Osiecki K, Lopez L, Goldstein H, Calderon TM, Berman JW (2006). CCL2/monocyte chemoattractant protein-1 mediates enhanced transmigration of human immunodeficiency virus (HIV)-infected leukocytes across the blood-brain barrier: a potential mechanism of HIV-CNS invasion and NeuroAIDS. J Neurosci.

[53] Schmidtmayerova H, Nottet HS, Nuovo G (1996). Human immunodeficiency virus type 1 infection alters chemokine beta peptide expression in human monocytes: implications for recruitment of leukocytes into brain and lymph nodes. Proc Natl Acad Sci U S A.

[54] Ellis RJ, Hsia K, Spector SA (1997). Cerebrospinal fluid human immunodeficiency virus type 1 RNA levels are elevated in neurocognitively impaired individuals with acquired immunodeficiency syndrome. HIV Neurobehavioral Research Center Group. Ann Neurol.

[55] Su ZZ, Kang DC, Chen Y, Pekarskaya O, Chao W, Volsky DJ, Fisher PB (2002). Identification and cloning of human astrocyte genes displaying elevated expression after infection with HIV-1 or exposure to HIV-1 envelope glycoprotein by rapid subtraction hybridization, RaSH. Oncogene.

[56] Fischer-Smith T, Croul S, Sverstiuk AE (2001). CNS invasion by CD14+/CD16+ peripheral blood-derived monocytes in HIV dementia: perivascular accumulation and reservoir of HIV infection. J Neurovirol.

[57] Aylward EH, Henderer JD, McArthur JC, Brettschneider PD, Harris GJ, Barta PE, Pearlson GD (1993). Reduced basal ganglia volume in HIV-1-associated dementia: results from quantitative neuroimaging. Neurology.

[58] Corona G, Vaccher E, Cattarossi G, Sartor I, Toffoli G (2005). Potential hazard of pharmacokinetic interactions between lopinavir-ritonavir protease inhibitors and irinotecan. AIDS.

[59] Ranki A, Nyberg M, Ovod V (1995). Abundant expression of HIV Nef and Rev proteins in brain astrocytes in vivo is associated with dementia. AIDS.

[60] Zhang K, McQuibban GA, Silva C (2003). HIV-induced metalloproteinase processing of the chemokine stromal cell derived factor-1 causes neurodegeneration. Nat Neurosci.

[61] Nath A (2002). Human immunodeficiency virus (HIV) proteins in neuropathogenesis of HIV dementia. J Infect Dis.

[62] Zheng J, Thylin MR, Ghorpade A (1999). Intracellular CXCR4 signaling, neuronal apoptosis and neuropathogenic mechanisms of HIV-1-associated dementia. J Neuroimmunol.

[63] Kaul M, Zheng J, Okamoto S, Gendelman HE, Lipton SA (2005). HIV-1 infection and AIDS: consequences for the central nervous system. Cell Death Differ.

[64] Gonzalez E, Rovin BH, Sen L (2002). HIV-1 infection and AIDS dementia are influenced by a mutant MCP-1 allele linked to increased monocyte infiltration of tissues and MCP-1 levels. Proc Natl Acad Sci U S A.

[65] Edinger AL, Mankowski JL, Doranz BJ (1997). CD4-independent, CCR5-dependent infection of brain capillary endothelial cells by a neurovirulent simian immunodeficiency virus strain. Proc Natl Acad Sci U S A.

[66] Albright AV, Shieh JT, Itoh T (1999). Microglia express CCR5, CXCR4, and CCR3, but of these, CCR5 is the principal coreceptor for human immunodeficiency virus type 1 dementia isolates. J Virol.

[67] Churchill MJ, Wesselingh SL, Cowley D, Pardo CA, McArthur JC, Brew BJ, Gorry PR (2009). Extensive astrocyte infection is prominent in human immunodeficiency virus-associated dementia. Ann Neurol.

[68] Childs EA, Lyles RH, Selnes OA (1999). Plasma viral load and CD4 lymphocytes predict HIV-associated dementia and sensory neuropathy. Neurology.

[69] Wiley CA, Achim C (1994). Human immunodeficiency virus encephalitis is the pathological correlate of dementia in acquired immunodeficiency syndrome. Ann Neurol.

[70] Brew BJ, Rosenblum M, Cronin K, Price RW (1995). AIDS dementia complex and HIV-1 brain infection: clinical-virological correlations. Ann Neurol.

[71] Mattson MP, Haughey NJ, Nath A (2005). Cell death in HIV dementia. Cell Death Differ.

[72] Griffin DE, Wesselingh SL, McArthur JC (1994). Elevated central nervous system prostaglandins in human immunodeficiency virus-associated dementia. Ann Neurol.

[73] Haughey NJ, Nath A, Mattson MP, Slevin JT, Geiger JD (2001). HIV-1 Tat through phosphorylation of NMDA receptors potentiates glutamate excitotoxicity. J Neurochem.

[74] Nath A, Geiger J (1998). Neurobiological aspects of human immunodeficiency virus infection: neurotoxic mechanisms. Prog Neurobiol.

[75] Corder EH, Robertson K, Lannfelt L, Bogdanovic N, Eggertsen G, Wilkins J, Hall C (1998). HIV-infected subjects with the E4 allele for APOE have excess dementia and peripheral neuropathy. Nat Med.

[76] Harezlak J, Buchthal S, Taylor M (2011). Persistence of HIV-associated cognitive impairment, inflammation, and neuronal injury in era of highly active antiretroviral treatment. AIDS.

[77] Price RW (1996). Neurological complications of HIV infection. Lancet.

[78] Haughey NJ, Cutler RG, Tamara A (2004). Perturbation of sphingolipid metabolism and ceramide production in HIV-dementia. Ann Neurol.

[79] Wiley CA, Soontornniyomkij V, Radhakrishnan L (1998). Distribution of brain HIV load in AIDS. Brain Pathol.

[80] Ances BM, Ellis RJ (2007). Dementia and neurocognitive disorders due to HIV-1 infection. Semin Neurol.

[81] Maschke M, Kastrup O, Esser S, Ross B, Hengge U, Hufnagel A (2000). Incidence and prevalence of neurological disorders associated with HIV since the introduction of highly active antiretroviral therapy (HAART). J Neurol Neurosurg Psychiatry.

[82] Chen P, Mayne M, Power C, Nath A (1997). The Tat protein of HIV-1 induces tumor necrosis factor-alpha production. Implications for HIV-1-associated neurological diseases. J Biol Chem.

[83] Brew BJ, Pemberton L, Cunningham P, Law MG (1997). Levels of human immunodeficiency virus type 1 RNA in cerebrospinal fluid correlate with AIDS dementia stage. J Infect Dis.

[84] Chang L, Ernst T, Leonido-Yee M, Walot I, Singer E (1999). Cerebral metabolite abnormalities correlate with clinical severity of HIV-1 cognitive motor complex. Neurology.

[85] Adle-Biassette H, Chrétien F, Wingertsmann L (1999). Neuronal apoptosis does not correlate with dementia in HIV infection but is related to microglial activation and axonal damage. Neuropathol Appl Neurobiol.

[86] Brabers NA, Nottet HS (2006). Role of the pro-inflammatory cytokines TNF-alpha and IL-1beta in HIV-associated dementia. Eur J Clin Invest.

[87] Brew BJ (2004). Evidence for a change in AIDS dementia complex in the era of highly active antiretroviral therapy and the possibility of new forms of AIDS dementia complex. AIDS.

[88] Chang L, Ernst T, Leonido-Yee M, Witt M, Speck O, Walot I, Miller EN (1999). Highly active antiretroviral therapy reverses brain metabolite abnormalities in mild HIV dementia. Neurology.

[89] Patel CA, Mukhtar M, Pomerantz RJ (2000). Human immunodeficiency virus type 1 Vpr induces apoptosis in human neuronal cells. J Virol.

[90] Toborek M, Lee YW, Flora G (2005). Mechanisms of the blood-brain barrier disruption in HIV-1 infection. Cell Mol Neurobiol.

[91] Ellis RJ, Deutsch R, Heaton RK (1997). Neurocognitive impairment is an independent risk factor for death in HIV infection. San Diego HIV Neurobehavioral Research Center Group. Arch Neurol.

[92] Conant K, McArthur JC, Griffin DE, Sjulson L, Wahl LM, Irani DN (1999). Cerebrospinal fluid levels of MMP-2, 7, and 9 are elevated in association with human immunodeficiency virus dementia. Ann Neurol.

[93] Turchan J, Pocernich CB, Gairola C (2003). Oxidative stress in HIV demented patients and protection ex vivo with novel antioxidants. Neurology.

[94] Sevigny JJ, Albert SM, McDermott MP (2004). Evaluation of HIV RNA and markers of immune activation as predictors of HIV-associated dementia. Neurology.

[95] Dore GJ, McDonald A, Li Y, Kaldor JM, Brew BJ (2003). Marked improvement in survival following AIDS dementia complex in the era of highly active antiretroviral therapy. AIDS.

[96] Ellis RJ, Gamst AC, Capparelli E (2000). Cerebrospinal fluid HIV RNA originates from both local CNS and systemic sources. Neurology.

[97] Shi B, Raina J, Lorenzo A, Busciglio J, Gabuzda D (1998). Neuronal apoptosis induced by HIV-1 Tat protein and TNF-alpha: potentiation of neurotoxicity mediated by oxidative stress and implications for HIV-1 dementia. J Neurovirol.

[98] Everall I, Luthert P, Lantos P (1993). A review of neuronal damage in human immunodeficiency virus infection: its assessment, possible mechanism and relationship to dementia. J Neuropathol Exp Neurol.

[99] Nath A, Anderson C, Jones M (2000). Neurotoxicity and dysfunction of dopaminergic systems associated with AIDS dementia. J Psychopharmacol.

[100] Lindl KA, Marks DR, Kolson DL, Jordan-Sciutto KL (2010). HIV-associated neurocognitive disorder: pathogenesis and therapeutic opportunities. J Neuroimmune Pharmacol.

[101] Wang GJ, Chang L, Volkow ND, Telang F, Logan J, Ernst T, Fowler JS (2004). Decreased brain dopaminergic transporters in HIV-associated dementia patients. Brain.

[102] Berger JR, Arendt G (2000). HIV dementia: the role of the basal ganglia and dopaminergic systems. J Psychopharmacol.

[103] Gisslén M, Price RW, Andreasson U (2016). Plasma concentration of the neurofilament light protein (NFL) is a biomarker of CNS injury in HIV infection: a cross-sectional study. EBioMedicine.

[104] Mollace V, Nottet HS, Clayette P, Turco MC, Muscoli C, Salvemini D, Perno CF (2001). Oxidative stress and neuroAIDS: triggers, modulators and novel antioxidants. Trends Neurosci.

[105] Bansal AK, Mactutus CF, Nath A, Maragos W, Hauser KF, Booze RM (2000). Neurotoxicity of HIV-1 proteins gp120 and Tat in the rat striatum. Brain Res.

[106] Johns Hopkins Hospital. https://health.usnews.com/best-hospitals/area/md/johns-hopkins-hospital-6320180.

[107] Justin Charles McArthur, M.B.B.S. M.B.B.S., M.P.H M.P.H (2021). Justin Charles McArthur, M.B.B.S., M.P.H. Johns Hopkins Medicine.

[108] (2015). UNESCO science report towards 2030. http://uis.unesco.org/sites/default/files/documents/unesco-science-report-towards-2030-part1.pdf.

